# Intramucosal Alpha‐Fetoprotein‐producing Early Gastric Cancer Without Vascular Invasion or Metastasis Diagnosed After Endoscopic Submucosal Dissection: A Case Report and Literature Review

**DOI:** 10.1002/deo2.70233

**Published:** 2025-11-03

**Authors:** Kohei Uyama, Hiroyoshi Iwagami, Riki Sakano, Yasuki Nakatani, Yoshito Uenoyama, Kazuo Ono

**Affiliations:** ^1^ Department of Gastroenterology and Hepatology Japanese Red Cross Wakayama Medical Center Wakayama Japan; ^2^ Department of Diagnostic Pathology Japanese Red Cross Wakayama Medical Center Wakayama Japan

**Keywords:** alpha‐fetoprotein‐producing early gastric cancer, endoscopic submucosal dissection, hepatoid adenocarcinoma, lymph node metastasis, vascular invasion

## Abstract

Alpha‐fetoprotein‐producing gastric cancer (AFPGC) is a rare histological subtype of gastric cancer that is often diagnosed at an advanced stage. In the present case, an elevated lesion was detected in the gastric antrum during upper gastrointestinal endoscopy screening and diagnosed as early gastric cancer of cT1a. The patient underwent endoscopic submucosal dissection (ESD), and pathological examination showed positive staining for AFP, leading to a diagnosis of AFPGC confined to the intramucosa (pT1a). An additional laparoscopic distal gastrectomy with lymphadenectomy was performed, which revealed no residual tumor and no lymph node metastasis. The patient has remained recurrence‐free for 4 years after the additional surgery. Intramucosal gastric cancer with AFP production is extremely rare, and limited data are available regarding the need for additional surgical resection after ESD. Notably, a previous case of metastatic recurrence in AFPGC of cT1a was reported. Therefore, treatment decisions should be made on the basis of a thorough discussion and the patient's full informed consent.

## 1 Introduction

Alpha‐fetoprotein‐producing early gastric cancer (AFPGC) is typically diagnosed at an advanced stage with high malignant potential and accounts for 1.2%–6.6% of all gastric cancers [1]. Compared with non‐AFPGC, AFPGC has significantly higher frequencies of lymph node metastasis, lymphatic invasion, and hepatic metastasis [2]. Therefore, metastatic risk is a concern for patients with AFPGC, even when the tumor is confined to the intramucosa.

Here, we report a case of AFPGC that was diagnosed after endoscopic submucosal dissection (ESD) and underwent additional surgical resection. No lymph node metastasis was observed, and the patient has remained recurrence‐free for 4 years after the additional surgery. At present, there is no consensus on whether additional surgery is necessary following the endoscopic curative resection of AFPGC. This case of AFPGC confined to the mucosa is considered rare and of clinical significance.

## 2 Case Report

**FIGURE 1:**
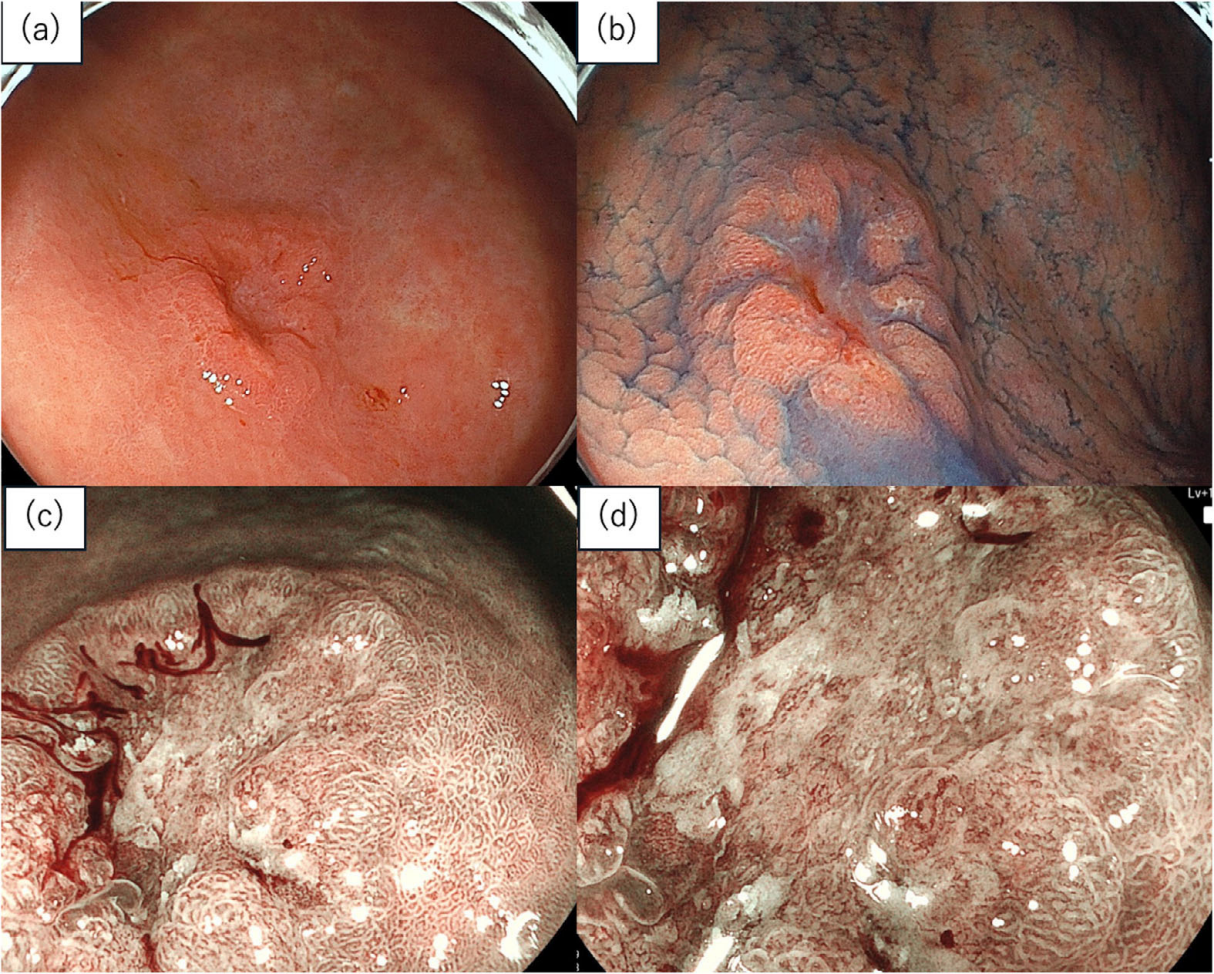
(a) Endoscopic examination revealed a 13 mm, reddish depressed lesion on the anterior wall of the antrum. (b) Indigo carmine dye spraying revealed the lesion as type 0‐IIc. (c) Magnifying endoscopic image showing that the micro surface pattern was absent. (d) Irregular vessels with caliber variation and tortuous course were observed.

**FIGURE 2:**
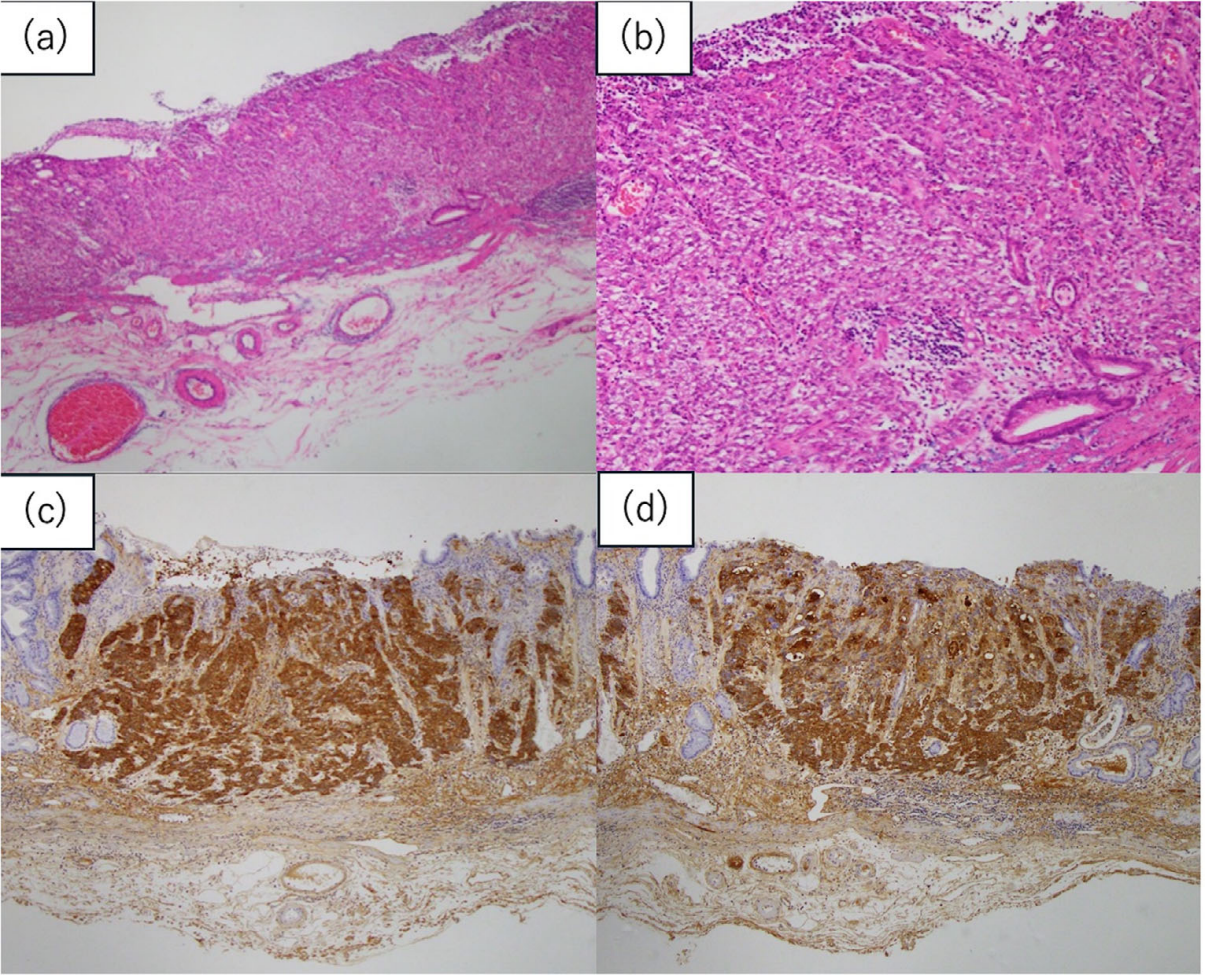
(a) Hematoxylin and eosin (HE) staining showing the solid proliferation of poorly‐differentiated adenocarcinoma with eosinophilic cytoplasm (×40). (b) Magnified image of 2a (HE×100). (c) Immunohistochemical staining for alpha‐fetoprotein was positive (×40). (d) Immunohistochemical staining for HepPar‐1 was positive (×40).

A 65‐year‐old man with a medical history of diabetes mellitus, hypertension, and prostate cancer underwent upper gastrointestinal endoscopy for screening at a local clinic hospital, which revealed a lesion in the gastric antrum. Analysis of the biopsied specimen indicated moderately to poorly differentiated adenocarcinoma, and the patient was referred to our hospital for further evaluation and treatment. He had a 45‐year history of smoking 20 cigarettes per day and consumed approximately 72 g of pure ethanol daily. He had no family history of gastric cancer, and *Helicobacter pylori* infection had been eradicated. He had no symptoms, and his physical examination was unremarkable. Laboratory findings were AFP 5.1 ng/mL, carcinoembryonic antigen 3.6 ng/mL, and carbohydrate antigen 19‐9 (CA19‐9) <0.5 U/mL, all within normal limits. Detailed endoscopic examination revealed a 13 mm, 0–IIc lesion on the anterior wall of the antrum (Figure 1a,b). Magnified blue laser imaging showed a clear demarcation line, an absent surface pattern, and an irregular microvascular pattern, consistent with gastric cancer (Figure 1c,d). Endoscopic ultrasound revealed preservation of the third layer. No evidence of lymph node or distant metastasis was observed on abdominal computed tomography. We diagnosed the lesion as cT1aN0M0, cStage IA, and ESD was performed. En bloc resection was achieved without any adverse events. Pathological examination revealed a 0–IIc, por > tub2, ly0, v0, HM0, VM0, pT1a, R0 lesion. Tumor cells contained eosinophilic cytoplasm and hepatocyte‐like morphology (Figure 2a,b), and immunohistochemical staining for AFP and HepPar‐1 was positive (Figure 2c). The tumor was diagnosed as AFPGC.

Given the potential risk of metastasis or recurrence [3]—even in the absence of lymphovascular invasion and with negative margins on the ESD specimen—the patient chose to proceed with additional surgical resection. This decision followed a detailed conversation with the attending doctor, during which previously reported cases of intramucosal AFPGC with metastasis were reviewed, including one with liver metastasis [9] and another with lymph node metastasis [10]. Taking these rare but documented outcomes into account, the patient opted for surgery to ensure a more definitive course of treatment. Laparoscopic distal gastrectomy with D1+ lymphadenectomy was performed. No lymph node metastasis was found. The patient has remained recurrence‐free for 4 years after surgery.

## 3 Discussion

AFPGC is a rare subtype of gastric cancer, accounting for 1.2%–6.6% of all cases, with histological types including adenocarcinoma with enteroblastic differentiation, hepatoid adenocarcinoma, and yolk sac tumor‐like carcinoma [1]. AFPGC tumors are often aggressive and detected at advanced stages, and cases of intramucosal lesions are extremely rare [4]. Previous studies have reported a high frequency of lymph node metastasis, lymphatic invasion, and liver metastasis in AFPGC, and the 5‐year survival rate is only 10%–20% [2].

This case represents a rare instance of intramucosal carcinoma, in which additional surgical resection confirmed the absence of lymph node metastasis. Moreover, this case may suggest long‐term survival of intramucosal AFPGC without vascular invasion or metastasis after endoscopically resection.

Most AFPGC tumors (86.1%) are located in the middle to lower third of the stomach. The predominant macroscopic type is 0–IIc in the early stage and type II in advanced stages. Detailed endoscopic features of early AFPGC have not been extensively reported. However, for early‐stage adenocarcinoma with enteroblastic differentiation, background mucosa typically shows severe atrophy (C‐3 or higher), and the color is reddish in all cases [5]. In a study of 14 cases, magnified endoscopy findings revealed a light blue crest in 81.8% of cases, a white opaque substance in 72.7%, and a white globe appearance in 9%; however, vessels within epithelial circle patterns were not observed [5]. None of the cases in the previous study were diagnosed as adenocarcinoma with enteroblastic differentiation as determined by biopsy. The difficulty in diagnosing tumors by biopsy may be because the tumor surface is predominantly composed of differentiated adenocarcinoma. Thus, diagnosing AFPGC solely using endoscopic or biopsy findings is considered difficult [5]. For these reasons, given that AFPGC is often diagnosed after resection, endoscopic treatment may be the initial approach.

Pathologically, hepatoid adenocarcinoma resembles hepatocellular carcinoma and is characterized by eosinophilic cytoplasm, solid sheet‐like growth, and AFP positivity. In a previous study of hepatoid adenocarcinomas, AFP was positive in 80% cases, SALL4 was positive in 47%, HepPar‐1 was positive in 69%, and GPC3 was positive in 56% [6]. This case was diagnosed as AFPGC based on AFP and HepPar‐1 positivity. The absence of further immunohistochemical analyses, including SALL4 and GPC3, may lessen the certainty of the diagnosis.

Even in a study limited to early‐stage cases, submucosal invasion occurred in 76% of AFPGCs, liver metastasis in 29%, and lymph node metastasis in 49%, indicating the need for cautious management even in early‐stage cancer [7]. Table 1 summarizes the previously reported cases of intramucosal AFPGC [3, 8, 9, 10]. Among the four cases, there was only one case without lymphatic invasion or metastasis (Case 1). Two were treated by endoscopic resection and observed without additional surgery (Cases 1 and 2). Case 1 remained recurrence‐free for 12 months, and Case 2 experienced nodal recurrence 1 year after endoscopic resection. In Case 3, surgical resection was performed as an initial treatment. A pathological diagnosis revealed intramucosal carcinoma with lymphatic invasion. The patient developed multiple liver metastases 3 years after surgery and died 77 months after surgery. In Case 4, surgical resection was performed as an initial treatment and revealed nodal metastasis. Among these four cases of intramucosal cancer, two showed lymph node metastasis and one had lymphatic invasion, suggesting that intramucosal AFPGC should not be managed in the same way as conventional differentiated adenocarcinomas. Our current case does not meet the guideline criteria for curative resection because AFPGC is classified as a special type of gastric cancer. Even if it is an intramucosal cancer, surgical treatment with lymph node dissection should be considered. Treatment plans must be determined with thorough informed consent. Although our patient has remained recurrence‐free for 4 years, the long‐term outcome data for intramucosal AFPGC are limited, and late recurrence has been reported; therefore, long‐term oncologic safety cannot be concluded from this single case. Further follow‐up is necessary to conclude.

**TABLE 1:** Summary of the previously reported cases of intramucosal alpha‐fetoprotein‐producing gastric cancer (AFPGC).

Case	Author	Sex/Age	Location	Macroscopic type	Tumor size	Histologic type	Depth	Lymph node metastasis	Liver metastasis	Treatment	Recurrence	Follow‐up period	Outcome
1	Horimatsu et al.	M/81	ND	0–IIa	70×60 mm	tub1	M	—	—	ESD	—	12 mo	Alive
2	Gokita et al.	M/70	L	0–IIc	12×6 mm	tub2	M	—	—	ESD	+	10 mo	Alive
3	Iwaya et al.	M/72	L	0–I	15×15 mm	hepatoid + tub	M+ ly (+)	—	+	Surgery	+	77 mo	Deceased
4	Sano et al.	M/76	L	0–IIc	10 mm	tub2	M	+	—	Surgery	—	68 mo	Deceased

Abbreviations: ly: lymphatic invasion; M (depth): mucosa; mo: months; ND: not described.

This case was confined to the mucosa and showed no evidence of lymphovascular invasion. Furthermore, the absence of lymph node metastasis was histologically confirmed by subsequent surgical resection. Current evidence is limited to a few case reports, but these findings can contribute to the accumulation of future evidence regarding whether additional surgical procedures after curative endoscopic resection are necessary.

In conclusion, reports of intramucosal AFPGC remain exceedingly rare, and there is no established consensus on whether additional surgical resection is warranted after curative endoscopic treatment. At present, therapeutic decisions must be based on the limited evidence available and reached through careful discussion with the patient. The continued long‐term follow‐up of this case, together with the accumulation and analysis of similar cases, will be crucial for guiding future management.

## Author Contributions

**Drafting the manuscript**: Kohei Uyama; **Provision of the patient's materials**: Riki Sakano; **Pathological evaluation**: Kazuo Ono; **Critical revision of the manuscript**: Hiroyoshi Iwagami, Yasuki Nakatani, and Yoshito Uenoyama. All authors have read and approved the final version of the manuscript.

## Conflicts of Interest

The authors declare no conflicts of interest.

## Funding

The authors received no specific funding for this work.

## Consent

Informed consent was obtained from the patient prior to inclusion in the study and publication of the manuscript.
